# Graft-implanted, enzyme responsive, tacrolimus-eluting hydrogel enables long-term survival of orthotopic porcine limb vascularized composite allografts: A proof of concept study

**DOI:** 10.1371/journal.pone.0210914

**Published:** 2019-01-24

**Authors:** C. Anton Fries, Shari D. Lawson, Lin C. Wang, Kai V. Slaughter, Praveen K. Vemula, Ashish Dhayani, Nitin Joshi, Jeffrey M. Karp, Rory F. Rickard, Vijay S. Gorantla, Michael R. Davis

**Affiliations:** 1 United States Army Institute of Surgical Research, San Antonio, TX, United States of America; 2 Royal Centre for Defence Medicine, ICT Centre, Birmingham, United Kingdom; 3 Center for Nanomedicine and Division of Engineering in Medicine, Department of Medicine, Brigham and Women’s Hospital, Boston, MA, United States of America; 4 Institute for Stem Cell Biology and Regenerative Medicine (inStem), UAS-GKVK Campus, Bangalore, India; 5 The School of Chemical and Biotechnology, SASTRA University, Thanjavur, India; 6 Harvard Medical School, Boston, MA, United States of America; 7 Wake Forest Institute for Regenerative Medicine, Wake Forest University School of Medicine, Winston Salem, NC, United States of America; 8 RESTOR Program, 59th MDW/ Science and Technology, Joint Base San Antonio, TX, United States of America; University of Toledo, UNITED STATES

## Abstract

**Background:**

Currently, patients receiving vascularized composite allotransplantation (VCA) grafts must take long-term systemic immunosuppressive therapy to prevent immunologic rejection. The morbidity and mortality associated with these medications is the single greatest barrier to more patients being able to receive these life-enhancing transplants. In contrast to solid organs, VCA, exemplified by hand or face transplants, allow visual diagnosis of clinical acute rejection (AR), directed biopsy and targeted graft therapies. Local immunosuppression in VCA could reduce systemic drug exposure and limit adverse effects. This proof of concept study evaluated, in a large animal forelimb VCA model, the efficacy and tolerability of a novel graft-implanted enzyme-responsive, tacrolimus (TAC)—eluting hydrogel platform, in achieving long-term graft survival.

**Methods:**

Orthotopic forelimb VCA were performed in single haplotype mismatched mini-swine. Controls (n = 2) received no treatment. Two groups received TAC hydrogel: high dose (n = 4, 91 mg TAC) and low dose (n = 4, 49 mg TAC). The goal was to find a dose that was tolerable and resulted in long-term graft survival. Limbs were evaluated for clinical and histopathological signs of AR. TAC levels were measured in serial blood and skin tissue samples. Tolerability of the dose was evaluated by monitoring animal feeding behavior and weight.

**Results:**

Control limbs underwent Banff Grade IV AR by post-operative day six. Low dose TAC hydrogel treatment resulted in long-term graft survival time to onset of Grade IV AR ranging from 56 days to 93 days. High dose TAC hydrogel also resulted in long-term graft survival (24 to 42 days), but was not well tolerated.

**Conclusion:**

Graft-implanted TAC-loaded hydrogel delays the onset of Grade IV AR of mismatched porcine forelimb VCA grafts, resulting in long term graft survival and demonstrates dose-dependent tolerability.

## Introduction

The life-changing reconstructive benefits and routine clinical utilization of VCA have been hampered by the risks related to lifelong, high-dose, multi-drug immunosuppression [1]. To date, uncontrolled acute rejection (AR) or chronic rejection (CR) has led to numerous graft losses [2,3]. Medication non-compliance is also a major contributor to preventable graft failure [4]. Tacrolimus (TAC), the mainstay drug in VCA, has a very narrow therapeutic range, with variable diurnal peaks and troughs after oral delivery [5]. Unlike solid organs, VCA offers unique opportunities for visual graft surveillance for clinical rejection as well as access to directed biopsies and graft targeted drug delivery [3,6,7].

Agents like TAC can be encapsulated in self-assembled hydrogels to create “enzyme-responsive depots”, that can be customized for on-cue spatiotemporal release in VCA tissues [8–10]. Our program has developed an injectable, enzyme-responsive delivery platform that provides on-cue release of TAC in VCA tissues in the presence of matrix metalloproteinases (MMPs), or other proteases in the extracellular milieu produced by graft infiltrating macrophages. MMPs (esp. MMP2 and MMP9) are critical mediators in AR and CR (vasculopathy) in solid organs. Suppressing early MMP (or other protease) driven immune events may be graft protective in VCA [6].

Prior work by team members in rodent limb VCA established the efficacy of the platform. A single-dose of TAC-laden hydrogel (7 mg TAC in 1 ml triglycerol monostearate [TGMS] gel), injected subcutaneously, allowed rejection-free limb transplant survival for more than 100 days with no additional systemic immunosuppression [10]. They have also demonstrated the utility of this platform in other diseases associated with over expression of MMPs and other enzymes [11,12]. This proof of concept study was designed to determine the tolerability and efficacy of the TAC delivery platform in a stringent, pre-clinical large animal (porcine), mismatched, orthotopic forelimb VCA model [13]. Specifically, we evaluated the tolerability and efficacy of two different doses of TAC-loaded TGMS hydrogel in porcine VCA. The goal was to identify a TAC dose that is tolerable and results in long-term graft survival. Given the relatively narrow therapeutic window for TAC, two doses that were close—49 mg and 93 mg—were investigated. VCA graft survival and episodes of acute rejection were evaluated. Tolerability of TAC hydrogel was determined by monitoring animal feeding behavior and weight.

## Methods

All experiments were performed at the Tri-Service Research Laboratories, United States Army Institute for Surgical Research, Fort Sam Houston, San Antonio, Texas. These were in accordance with a protocol independently reviewed and approved by the Institutional Animal Care and Use Committee (IACUC) of the Tri-Service Research Laboratory.

### Animals

Single haplotype mismatched Yucatan mini-pigs (Sus scrofa domesticus) (Sinclair Bio Resources LLC, Columbia, MO), served as donors and recipients for VCA procedures. All animals were housed and maintained in accordance with IACUC guidelines. Procedures were in compliance with American Association for the Accreditation of Laboratory Animal Care (AALAC) recommendations and the principles set forth in the National Institute of Health Publication, ‘Guide for the Care and Use of Laboratory Animals’ and the Animal Welfare Act of 1966, as amended. Humane endpoints were used in this study to determine time of euthanasia, out-with the protocol endpoints of grade IV limb rejection, or reaching the end of the protocol duration (100 days); otherwise euthanasia was performed on reaching these protocol end-points. Animal death was not an endpoint of the study. Euthanasia was by means of intravenous Sodium-Pentobarbitol, 100mg/kg, via ear vein. Animals were reviewed daily by the veterinary technicians and research team and as required by the staff veterinarian of the TSRL. The analgesic regimen required was Buprenorphine SR (ZooPharm, Windsor, CO) 0.15mg/kg every 72 hours and ketoprofen (Fort Dodge animal health, New York, NY) 3mg/kg immediately post-operatively and then on an as required basis at the discretion of the attending veterinarian. All research team members had completed the American Association for Animal Laboratory Sciences training courses in Pain Recognition and Alleviation in Laboratory Animals and Euthenasia of Research Animals: AVMA Guidelines. In total 10 animals were utilized in this protocol of which none were found dead, two were euthanized prior to study end points. In both of these cases this was due to failure to thrive of the animals manifest by loss of body weight. In all cases euthanasia was performed immediately animals had reached study endpoints.

### Orthotopic porcine forelimb transplantation

**Fig 1:**
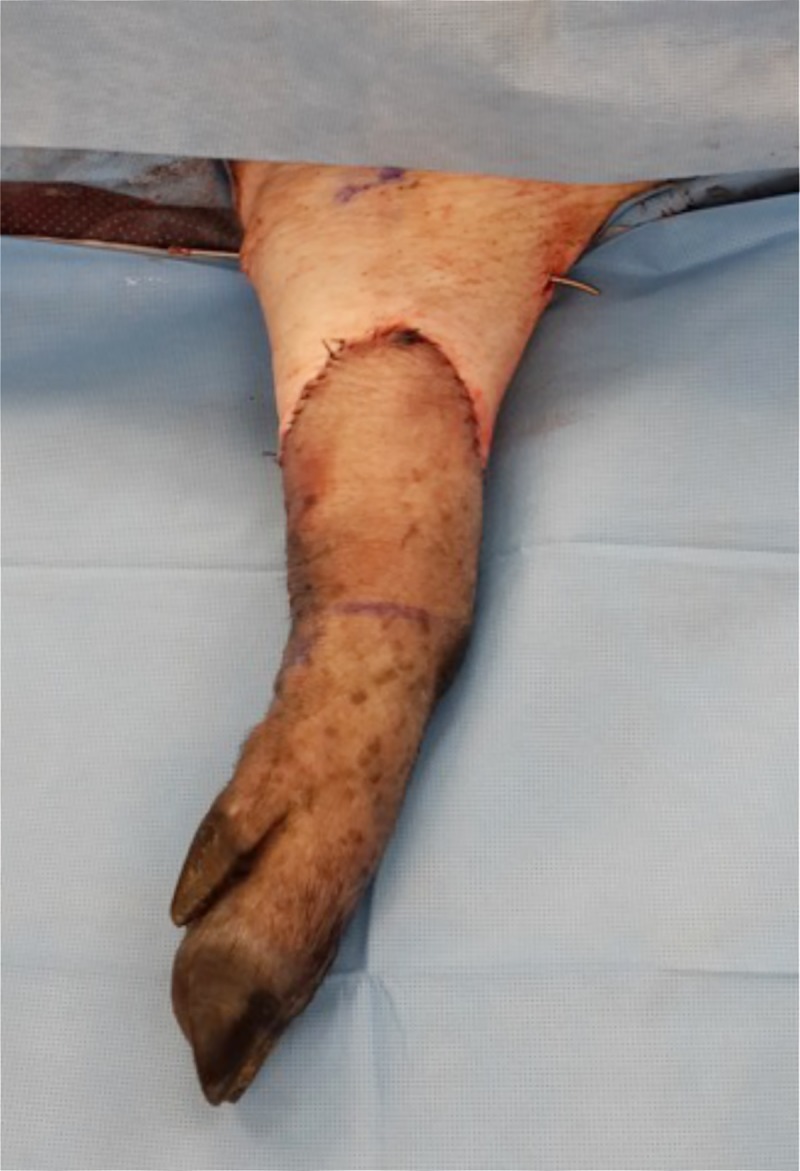
Orthotopic forelimb allotransplantation. Transplant recipients can mobilize immediately after recovery in a surgical cast that is fixed with a single Steinmann pin.

This protocol utilized an orthotopic, fully weight-bearing porcine forelimb VCA model that was developed by our group and previously published [13]. Salient procedural details are summarized as follows:

Anesthesia was induced and maintained by isoflurane following premedication with intramuscular ketamine. Subjects were positioned supine with the left forelimb in abduction. Two teams operated simultaneously to prepare donor and recipient. The left forelimb was dissected at its mid-point via a “fish-mouth” skin incision. The neurovascular bundle, containing the brachial artery and associated vena comitantes and median nerve was exposed, ligated and divided. Due attention was given to adequacy of length of the neurovascular pedicle and tendons in order to achieve a tension free neuro-vascular repair and physiological tendon balancing following transplantation. An osteotomy was performed at the midpoint of the radio-ulna on the donor and recipient and rigid fixation was accomplished with two weight bearing six-hole 3mm plates and tri-cortical locking screws. All neurovascular structures were microsurgically coapted and tendon repairs performed using standard techniques. The skin was closed without tension and leg splinted in a plaster cast anchored by a single Steinmann pin to prevent slippage of the cast (Fig 1).

### Immunological mismatch

Donor and recipient pairs were selected across a standardized immunologic mismatch [14]. For clinical relevance, a mismatch was sought equivalent to an un-related deceased donor (one HLA mismatch). Four distinctive porcine leukocyte antigen (SLA) haplotypes, locally designated as “w”, “x”, “y” and “z”, were characterized in the Yucatan miniature pigs breeds [14]. A crossover haplotype, designated “q”, consisting class I of “w” and class II of “z”, was also detected in the breed. The SLA genotype of the Yucatan pigs used in this study was verified at three class I (SLA-1, -2, -3) and three class II (DRB1, DQB1 and DQA) genes using the low-resolution (Lr) PCR-SSP (sequence-specific primer) typing assays as described [15].

### Groups and interventions

Three animal groups were investigated in the study. Group 1 (Controls, n = 2), received no treatment. Group 2 (Experimental group, n = 4) received high dose TAC hydrogel, 91mg, per limb. Group 3 (Experimental group, n = 4) received low dose TAC hydrogel, 49 mg, per limb. Doses were estimated based on extrapolations on a per-weight basis using allometric calculations from prior published rat hind limb studies [10].

### Preparation of TAC hydrogels

TAC eluting, self-assembled, amphiphilic triglycerol monostearate (TGMS) hydrogels were prepared by Dr Karp’s laboratory at Brigham and Women’s hospital, Boston, MA [9]. Encapsulation of TAC to form TGMS-TAC hydrogels was achieved by heating TGMS (10%w/v) and 7 mg of TAC in DMSO/water (1:4 v/v) in a glass scintillation vial to 60–80°C until dissolution resulting in TAC concentrations of 7 mg/ml. The vial was allowed to cool until gelation had occurred. The resultant hydrogel containing 7 mg/ml TAC was loaded in individual 1 ml syringes. These were stored in refrigerated conditions (4°C) until used and allowed to reach room temperature prior to injection.

### Administration of TAC hydrogel and dosing protocol

Immediately prior to skin closure the TGMS-TAC hydrogel was injected through a 19-G needle into the loose connective tissue in the sub-dermal plane of the forelimb VCA. Animals received TAC hydrogels in two dosing regimens: high dose (Group 2, n = 4, 91 mg TAC) and low dose (Group 3, n = 4, 49 mg TAC). Each injection was in the form of a 1ml aliquot containing 7mg of TAC. The limb was divided into identically sized quadrants, each of which received the total dose in 1ml single split-dose injections. The goal was to achieve a uniform distribution of drug in the VCA tissues. The skin was then closed using interrupted 3–0 vicryl sutures to the dermal layer and 4–0 nylon to the skin.

### Clinical and histopathologic assessment of rejection

Grafts were monitored daily for signs of acute rejection and histopathologically by skin biopsy on post-operative days one, four, seven and then weekly thereafter until the end point of study (the development Banff Grade IV rejection). The primary clinical and histopathologic diagnosis of rejection was based on previously described Banff Classification of VCA [16–18]. Clinically grafts were monitored for a consistent progression of rejection from erythema, macule formation, blistering, desquamation of the epidermis and finally frank necrosis. Animals were sedated with intramuscular 4-6mg/kg Telazol (tiletamine hydrochloride and zolazepam hydrochloride combination, Zoetis, Parsippany, NJ). The casts were removed and limbs examined and photographed. Two representative 4 mm punch skin biopsies were taken from limb areas most affected clinically by rejection and frozen in liquid nitrogen (for tissue TAC levels) or fixed in 10% buffered formalin and paraffin embedded. All were stained with hematoxylin-eosin (H&E; Fischer Scientific, Fair Lawn, NJ) after rehydration with serial xylene, ethyl alcohol, and deuterated water rinses. Slides were evaluated by an independent, blinded, veterinary pathologist with transplant experience. The staff veterinarian at the TSRL performed necropsies on all subjects following euthanasia once study end-points had been reached to further evaluate evidence of drug toxicity.

### Assessment of TAC levels in whole blood and skin of VCA

Auricular vein blood sampling was performed regularly (every 3–4 days for the first two weeks followed by every 6–10 days after till end point). Whole blood levels of TAC, were analyzed using liquid chromatography mass spectroscopy (LC–MS/MS). Forelimb skin biopsies were collected simultaneously with blood samples for tissue TAC level measurement. Skin biopsies of the VCA were homogenized and TAC extracted with methanol. The methanolic solution was evaporated and residue was reconstituted with blood/plasma and analyzed by LC–MS/MS. Blood and tissue drug levels were calculated and expressed as ng/ml or ng/ml of homogenate. Samples were vortex mixed for 30s with a mixture of methanol and ZnSO_4_ (70:30, v/v). Ascomycin was used as an internal standard for TAC. After centrifugation, the supernatant was put in an auto sampler for injection into the system. Drug was eluted on C18-reversed phase column (150 mm, 3.0 mm; 5μm) by a mixture of water and ammonium acetate solution (80:20 v/v). Intra-assay and inter-assay imprecisions were acceptable (<10%), and mean absolute recovery was 89%. This method was validated in the range of 2 – 40ng/ml for TAC with the lower limit of quantification (LLQ) set at 2ng/ml for TAC with an acceptable precision (CV<15%). Each sample was analyzed in three replicates [19].

### Data analysis

Pharmacokinetic profiles and parameters were evaluated using Graph pad prism 6 and Winnonlin 6. Systemic exposure (C_max_) and local tissue concentrations were measured after drug encapsulated hydrogel administered directly into the graft. For statistical analysis, mean defect areas and standard deviations were calculated and compared among groups by a 3 x 4 (group by time post-op), and two-way analysis of variance (ANOVA). Inter-group differences were assessed by Bonferroni multiple-comparison test (SPSS v12). Mean differences were considered significant if p < 0.05. In all experiments VCA survival between groups was compared by ANOVA or Turkey-Kramer post hoc test where appropriate. When only two comparisons could be made, an unpaired two-sided t-test was used. Continuous variables were expressed as mean ± SEM. For the ANOVA outcomes, 80% power and 5% significance were used to find a 25% difference in outcomes. Survival analysis and differences in survival probabilities along with their standard errors were reported using log rank (Mantel-Cox) statistics.

## Results

### High dose and low dose hydrogels prolong graft survival compared to controls

**Fig 2:**
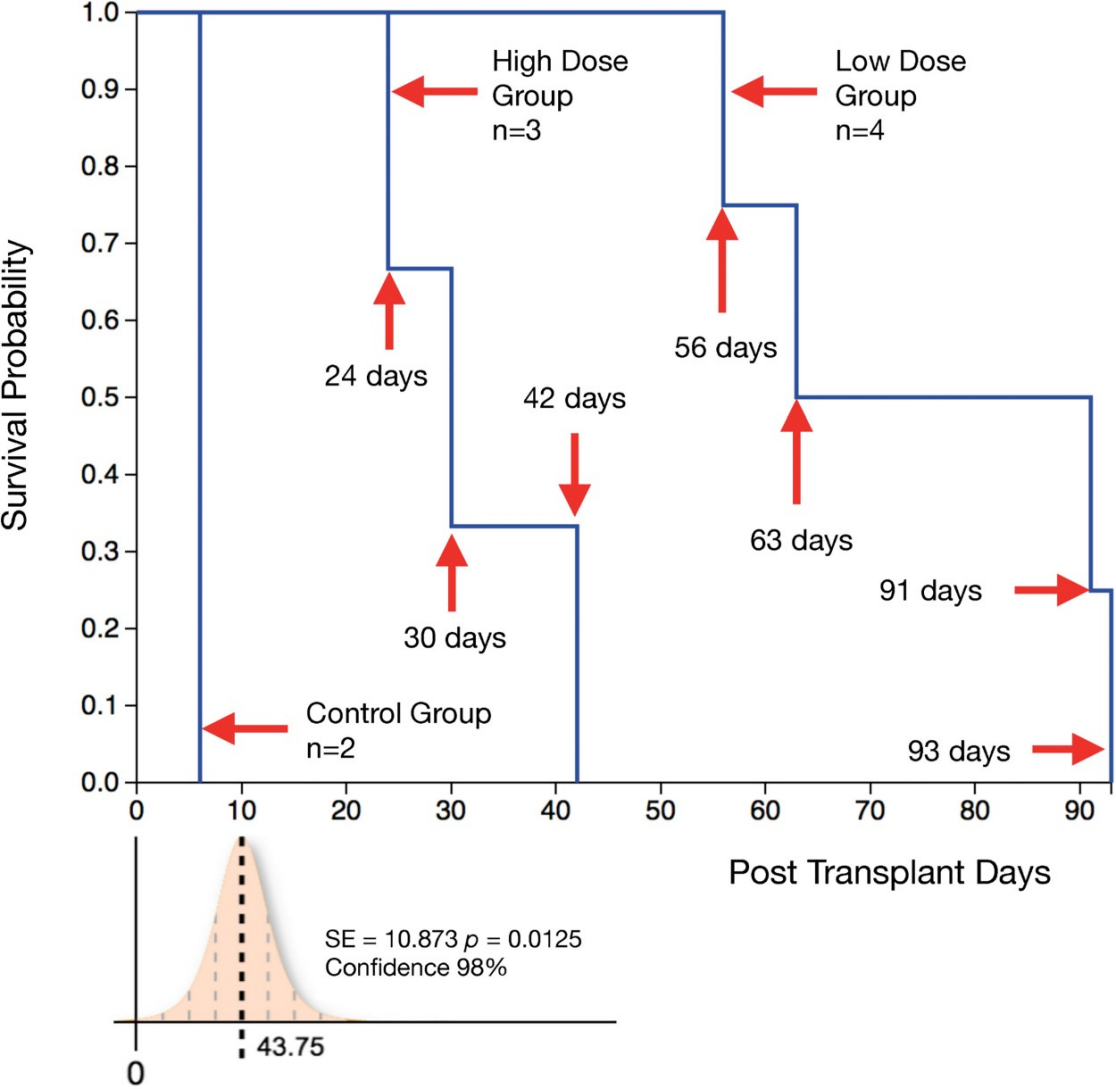
Kaplan-Meier survival plot of time to reaching Grade III rejection in transplanted limb. The hazard rate for AR differs between the high dose and low dose group (log-rank test; z = 2.57, p = 0.0101, Confidence—98%). There is a survival difference between high dose group (death censored) and low dose group (p = 0.0125) (high dose animals required to be euthanized with non- rejecting grafts due to failure to thrive).

Untreated Group 1 controls (n = 2) reached Grade IV AR at post-operative day (POD) 6 and 7 respectively. In Group 2 (n = 4; high dose TAC hydrogel, 91mg per limb) one animal was excluded from study due to flap failure on POD 1. Three animals that were followed up for the study showed prolonged graft survival without onset of Grade IV rejection compared to controls. However, they failed to thrive with poor feeding and weight loss, requiring early euthanasia at varying time points (POD 24,30,42). Pancreatitis was demonstrated post-mortem in these animals. Group 3 animals (n = 4; low dose TAC hydrogel, 49mg per limb) showed prolonged graft survival to onset of Grade IV rejection (POD 56,63,91,93). The survival difference between the high dose group (death censored) and low dose group was statistically significant (p = 0.0125); the high dose animals required to be euthanized with non-rejecting grafts due to failure to thrive (Fig 2).

### Drug release from hydrogels coincides with graft immune events and macrophage activity

**Fig 3:**
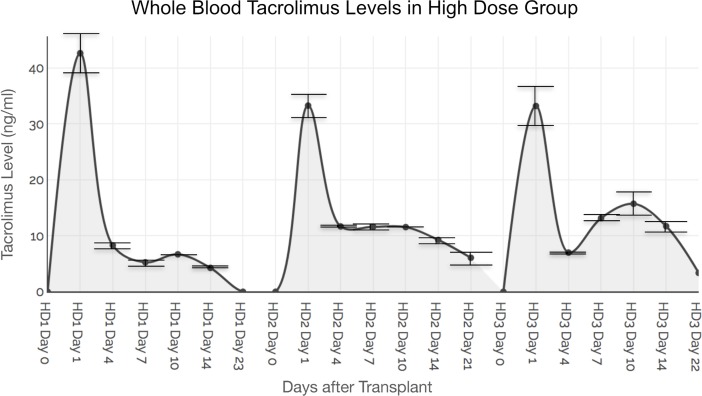
Whole blood tacrolimus levels in high dose group. Animals receiving hydrogels containing 91 mg (total dose) of TAC demonstrated a burst release of TAC (ranging between 30 and 40 ng/ml) at POD 1 that coincided with macrophage activation secondary to inflammatory events from surgical trauma and ischemia-reperfusion injury. A secondary spike of TAC release was observed at around POD 10 in all animals that possibly coincides with onset of AR events in the VCA. Lower panel demonstrates mean TAC levels coinciding with macrophage-mediated graft immune events. Standard deviations (SD) of TAC levels in triplicate whole blood samples are shown.

Whole blood TAC levels (averaged from triplicate samples, mean +/- SD) in the three Group 2 animals were: 42.6 ng/ml +/- 2.73 ng/ml on POD 1 reducing to 4.27 +/- 0.14 ng/ml on POD 23; 33.35 +/- 4.74 ng/ml on POD 1 reducing to 6.08 +/-0.22 ng/ml on POD 21; and 33.18 +/- 2.95 ng/ml on POD 1 reducing to 3.37 +/- 0.18 ng/ml on POD 22. Fig 3 shows whole blood levels and of systemic TAC values of triplicate samples at each time point.

**Fig 4:**
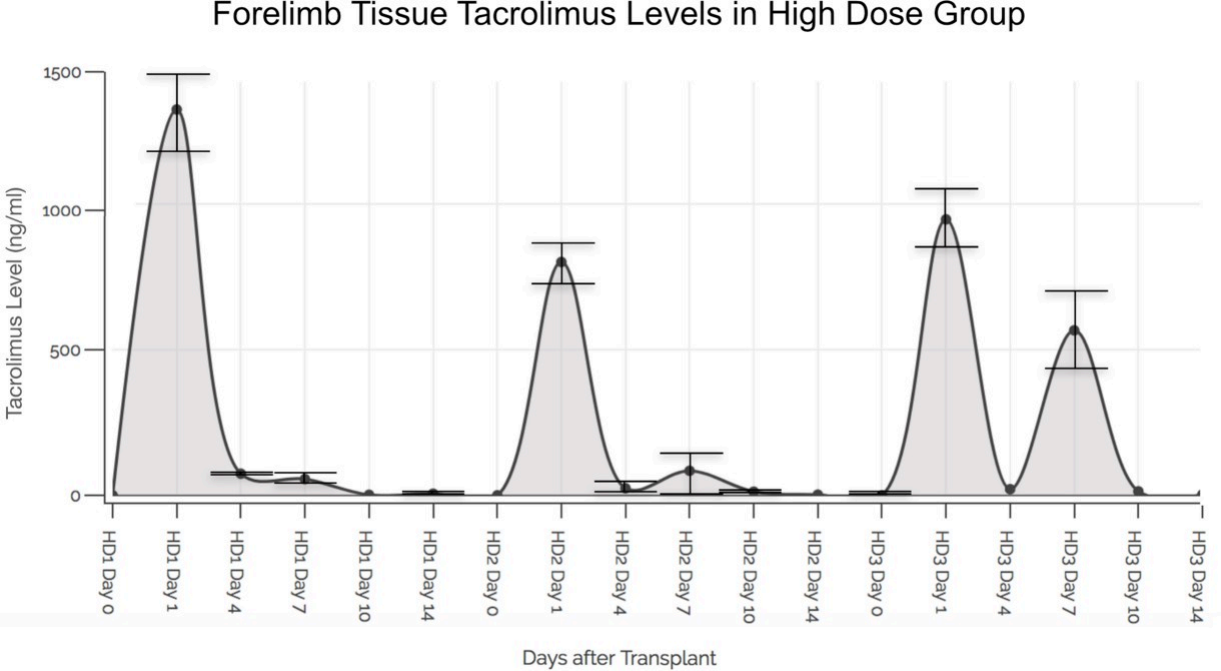
Tacrolimus levels in forelimb VCA skin tissue in high dose group. Animals receiving hydrogels containing 91 mg (total dose) of TAC demonstrated a burst release of TAC (ranging between 500 and 1500 ng/gm) in forelimb skin tissue at POD 1 that coincided with macrophage activation secondary to inflammatory events from surgical trauma and ischemia- reperfusion injury. A secondary spike of TAC release was observed at around POD 7 in all animals that possibly coincides with onset of AR events in the VCA. Mean TAC levels coinciding with macrophage-mediated graft immune events are shown. Standard deviations (SD) of TAC levels in triplicate tissue samples are shown.

The tissue TAC levels (in triplicate samples) in the skin in the three Group 2 animals were as follows: 1322.74 +/- 162.96 ng/ml on POD 1 reducing to 5.60 +/- 0.24 ng/ml on POD 14; 799.51 +/- 9.43 ng/ml on POD 1 reducing to 2.16 +/- 0.21 ng/ml on POD 14; and 945.88 +/- 6.28 ng/ml on POD 1 reducing to 2.93 +/- 1.11 ng/ml on POD 14. The levels of TAC in the graft tissues versus the whole blood in the same animal at similar time points of analysis were significantly higher (ranging from 100 to 1000-fold, p > 0.001 to p > 0.0001) (Fig 4).

**Fig 5:**
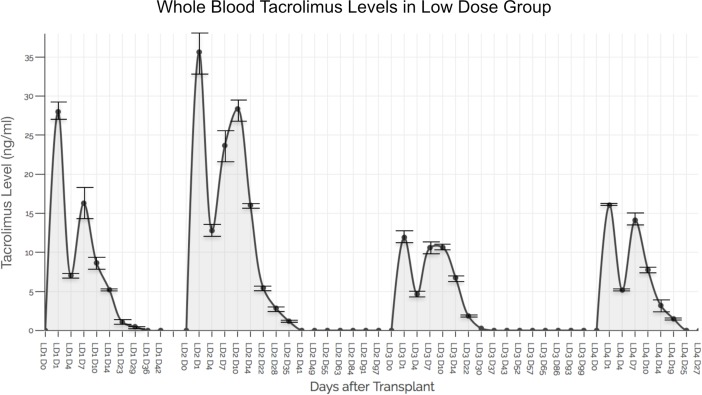
Whole blood tacrolimus levels in low dose group. Animals receiving hydrogels containing 49 mg (total dose) of TAC demonstrated a burst release of TAC (ranging between 10 and 35 ng/ml) at POD 1 that coincided with macrophage activation secondary to inflammatory events from surgical trauma and ischemia-reperfusion injury. A secondary spike of TAC release was observed between POD 7–10 in all animals that possibly coincides with onset of AR events in the VCA. Mean TAC levels coinciding with macrophage- mediated graft immune events are shown. Standard deviations (SD) of TAC levels in triplicate whole blood samples are shown.

Whole blood TAC levels (in triplicate samples) in the four Group 3 animals were as follows: 27.98 +/- 1.48 ng/ml on POD 1 reducing to 0.48 +/- 0.15 ng/ml on POD 29 and to undetectable thereafter until end point POD 42; 35.62 +/- 1.19 ng/ml on POD 1 reducing to 1.19 +/- 0.15 ng/ml on POD 35 and to undetectable thereafter until end point POD 97; 11.91 +/- 0.5 ng/ml on POD 1 reducing to 0.27 +/- 0.25 ng/ml on POD 30 and to undetectable thereafter until end point POD 99; 16.05 +/- 0.77 ng/ml on POD 1 reducing to 1.48 +/- 0.24 ng/ml on POD 19 and to undetectable thereafter until end point POD 27 (Fig 5).

An initial spike in TAC release was observed in Group 2 in whole blood samples on POD 1 after surgery (coincident with macrophage activity in surgical inflammation and reperfusion injury) [20]. Another spike was observed at POD 7 or 10 after surgery (coincident with macrophage activity in AR) (Fig 3) [21]. These findings are mirrored in the results of the tissue samples at the same time-points (Fig 4).

Consistent with findings in Group 2, Group 3 animals demonstrated an initial spike in TAC release in whole blood samples on POD 1 after surgery (coincident with macrophage activity in surgical inflammation and reperfusion injury) and at POD 7 or 10 after surgery (coincident with the timing of AR in control animals) (Fig 5).

**Fig 6:**
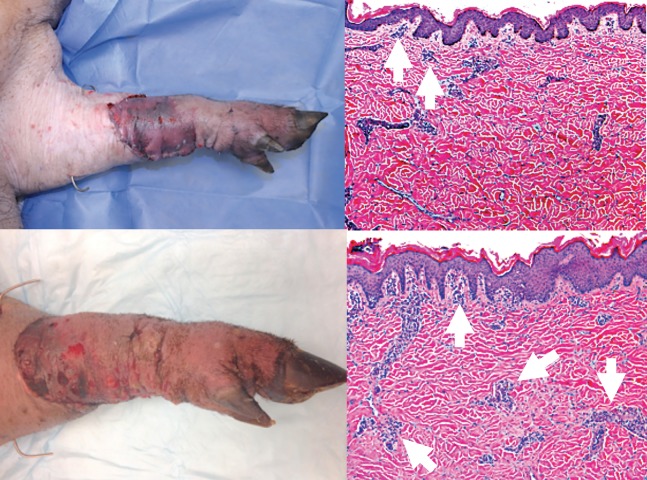
Clinical and histopathologic assessment of rejection. Top left panel: Banff Grade 1 AR (clinical picture) Top right panel: Histopathology of Grade 1 AR showing mild perivascular infiltration (arrows). No involvement of overlying epidermis. Bottom left panel: Banff Grade 2 AR (clinical picture) Bottom right panel: Histopathology of Grade 2 AR showing perivascular inflammation with/without mild epidermal or adnexal involvement (arrows). No epidermal dyskeratosis or apoptosis observed.

**Fig 7:**
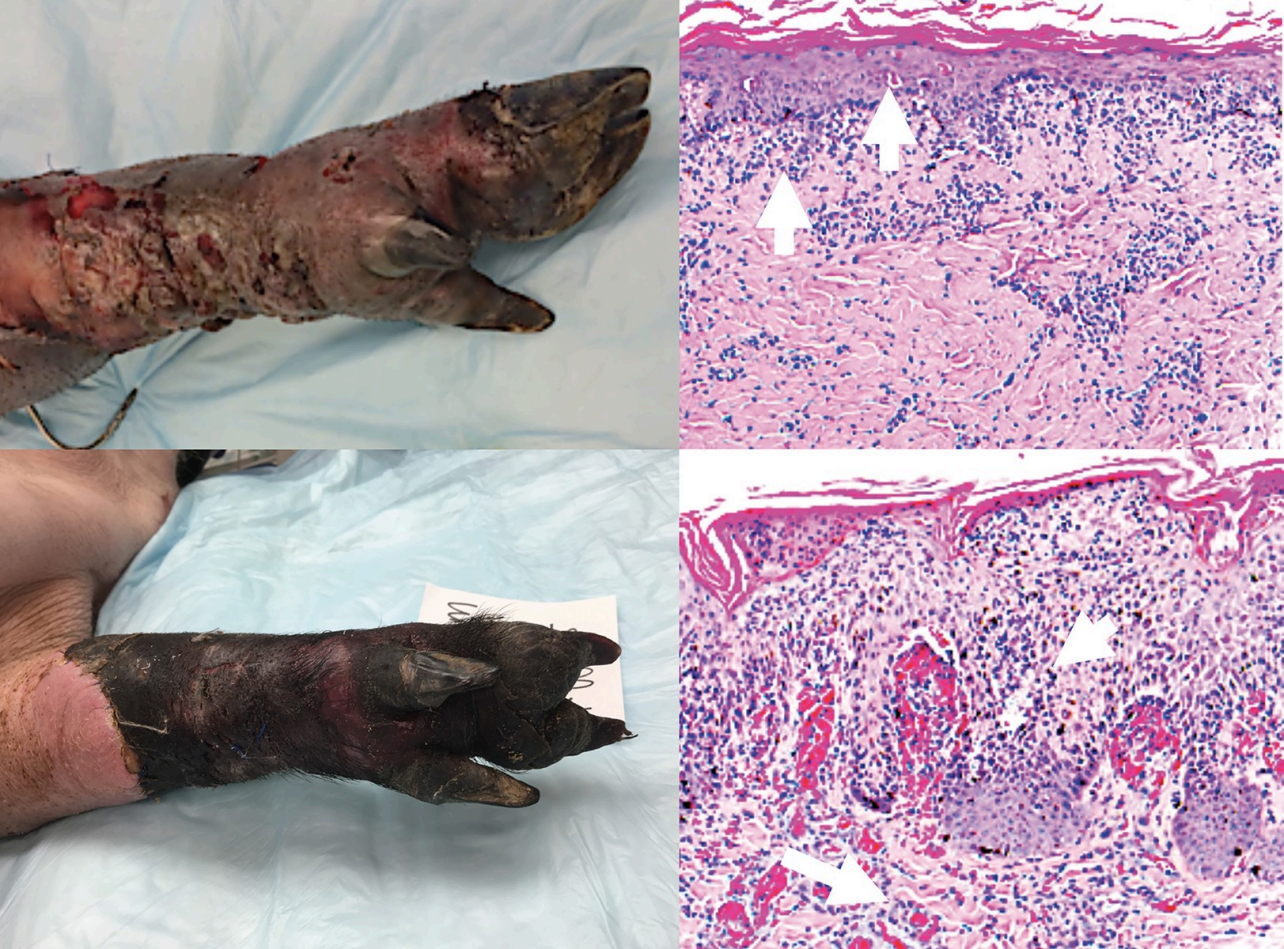
Clinical and histopathologic assessment of rejection. Top left panel: Banff Grade 3 AR (clinical picture) Top right panel: Histopathology of Grade 3 AR showing dense inflammation and epidermal involvement with apoptosis, dyskeratosis, and/or keratinolysis (arrows). Bottom left panel: Banff Grade 4 AR (clinical picture) Bottom right panel: Histopathology of Grade 4 AR showing necrotizing acute rejection. Frank necrosis of epidermis and presence of microvascular thrombi in deep dermal capillaries.

Figs 6 and 7 show representative images of clinical and histopathological manifestations of rejection in this study. According to the Banff classification of skin containing composite tissue allografts manifestations of acute cell-mediated rejection includes immune cell infiltration of the skin (this may be neutrophils and / or lymphocytes) to the dermis and epidermal and / or adnexal involvement [17].

## Discussion

Despite evolving clinical experience and progress in the understanding of the biology of VCA, one of the main factors preventing wider acceptance and routine clinical application are the associated adverse effects of long-term immunosuppression [22]. Since most VCA are non-life-saving procedures, the risks and toxicity of immunosuppression must be carefully balanced against their potential life enhancing benefits [23].

TAC remains the mainstay in the majority of VCA drug regimens [5]. TAC is a calcineurin inhibitor with a very narrow therapeutic range (range of exposure between the therapeutic threshold and the toxic threshold). Furthermore its pharmacokinetics result in blood level fluctuation, or diurnal peaks and troughs and intra or inter-patient blood level variability after oral delivery. Variable intestinal absorption or skipped doses due to non-compliance may lead to recurrent under immunosuppression and increased risk of AR or CR [24]. Attempts to restore trough levels to the therapeutic range may result in over immunosuppression resulting in supra-threshold peaks with risks such as nephrotoxicity, malignancy and opportunistic infection [25]. Consistent and reliable maintenance of patients within a therapeutic range is thus extremely challenging. Finally TAC blood levels do not proportionally correlate with graft tissue drug concentrations and there is significant inter-patient and intra-patient variability in drug exposure at comparable doses [7].

Oral administration of TAC in VCA is associated with extensive first-pass metabolism in the liver, greatly reducing its bioavailability due to actions of enzymes of the gastrointestinal lumen and lining, bacterial enzymes, and hepatic enzymes. Combined with possible renal clearance, only a small percentage of the drug typically reaches target graft tissues. The ratio of systemic versus local graft exposure is thus very high. Consequently, large and repeated dosing is often necessary.

Unlike solid organs, VCA tissues are accessible for visual monitoring and local intervention, such as topical therapies. It is thus possible to administer immunosuppressants locally to the graft, avoiding or minimizing systemic immunosuppression [3,7]. Recent innovation in bioengineering, nanotechnology, and regenerative medicine has enabled the development of a hydrogel system that can be embedded in transplanted grafts [26–28]. Such site-specific graft immunosuppression could facilitate long term graft survival while minimizing systemic immunosuppression and reducing the number of systemic drugs required [6,7]. TAC-loaded hydrogel can be injected subcutaneously or intramuscularly, to act as a drug depot that is enzyme responsive. The hydrogel can be disassembled by MMPs produced by macrophages including Langerhans cells and dermal dendritic cells to release TAC [29]. Such a system shows minimal drug release in normal physiological conditions but increased drug release when there is immune activity in the VCA tissues, resulting in prolonged efficacy of the gel. Most importantly, such graft embedded hydrogels may improve safety, efficacy, and patient compliance.

Our porcine model of orthotopic forelimb VCA provides the requisite stringency to investigate the efficacy of TAC-loaded TGMS hydrogel in a large animal model. Swine are relatively docile, economical and have very similar anatomy and tissue composition to humans, making them an optimal model for VCA. Also, the immune responses in this model are similar to those observed in humans [30].

In this proof of concept, exploratory study, a single dose of both low dose (49mg / forelimb graft) and high dose (91mg / forelimb graft) TAC hydrogels achieved long-term survival, ranging from 24 days to 93 days. The low dose was better tolerated that the high dose, which resulted in weight loss and poor feeding, in these cases pancreatitis was diagnosed post-mortem. Notably, these results were achieved in the absence of any systemic immunosuppression or antibody induction as in the clinical scenario. This is the first time such long-term VCA survival has been demonstrated in a pre-clinical large animal model with a graft implanted TAC delivery platform.

Limitations of the study that merit further elucidation in future work are acknowledged. Animal numbers were chosen with adherence to the principle of ‘reduction’ of live subjects to that which would enable demonstration of proof of concept. Additional groups, including controls with standard systemic immunosuppression (tacrolimus, mycophenolate mofetil +/- corticosteroids), for example would have added power to the protocol. Attempts at using linear regression models for correlating Banff grades of AR with graft survival in animals were constrained by the low animal numbers. This type of information can be extremely valuable as this drug delivery platform is developed for clinical application.

Repeated biopsies in the VCA graft could have triggered iatrogenic inflammation with macrophage trafficking and activation. We thus did not rely on immunohistochemical evaluation of macrophage specific markers (such as CD68) on biopsy samples as macrophage infiltration could occur due to the biopsy-induced inflammation. Rather, an assessment of T-cell infiltration and correlation of severity and location of lymphocytic infiltration with standardized grading systems such as the Banff Score of AR was performed. Whilst no animals developed signs of systemic sepsis the presence of a Steinman pin for retention of the cast in the initial phases may also have triggered some TAC release. These factors could have caused non-specific release of TAC, potentially leading to premature drug exhaustion in the gels with breakthrough AR and accelerated graft loss. The desire to perform more regular skin biopsies was tempered by this concern. The lack of a depletional induction regimen as used clinically sets a higher burden for success on the hydrogel drug delivery system [31–33]. Adjunctive systemic immune suppression was not included on this protocol to prevent confounding the effects of the graft embedded platform, however it is recognized that in clinical practice these will likely be combined [34]. Although Group 2 animals receiving the higher dose TAC suffered from morbidity as compared to Group 3, the whole blood concentrations (Cmax of TAC) as well as time (in days) to baseline (standard error 3.536, confidence 99% and p = 0.0492) were not significantly different between the two groups (Figs 2 and 3). A bioequivalence study of the two doses and the time points of testing during the follow was not performed, thus not allowing for an area under curve (AUC) determination with each dosing regimen. Future studies will focus on correlation of Cmax with the AUC to develop bioequivalence of dosing regimens. Cmax/AUC measurements could address intra-subject variations and pharmacokinetics of the gel platform in VCA.

It was found that increase in tissue levels and whole blood levels of TAC coincided in timing with inflammation associated with the surgical trauma or rejection responses as confirmed by biopsy. It is also possible that tissue levels of TAC could have varied based on the site of skin biopsy and subsequent inflammation. This is because of variables such as amount of drug released in the vicinity of the biopsy (macrophage activity secondary to trauma induced inflammation can fluctuate across the graft as AR can be heterogeneous) and differing fat content in the skin; TAC is lipophilic and porcine tissues have variable adipose tissue concentration depending on site [3, 35–37]. We were constrained in our analysis of macrophage migration and activation patterns due to the lack of availability of non-invasive cell tracking methods as well as in vivo cellular markers of macrophage activation in the porcine model. However, as the biopsies were taken at the site of most severe rejection, it is considered that higher levels of TAC here would be representative of the response. All these factors imply that the sensitivity and specificity of the TAC hydrogel delivery system as well as the measurement and monitoring methodology of graft delivered immunosuppression in VCA applications has to be optimized. An important goal is to restrict TAC release to the specific setting of AR and not in response to non-specific (such as infection) or iatrogenic inflammation (such as punch biopsy). Repeated dosing (every 20–30 days) may improve antirejection efficacy of this platform by improving bioavailability and bioactivity of TAC. These efforts are ongoing in our laboratories.

Taken together, an in-situ, graft implanted, immunosuppressive system as proposed holds promise towards long-term graft survival and improved patient quality of life in VCA. Allograft targeted immunosuppressive strategies therefore deserve further investigation to reduce risk and expand the broader clinical benefits of VCA.

## Supporting information

S1 Table High dose animals (Group 2) blood tacrolimus levels. (XLSX)

S2 Table High dose animals tissue (Group 2) tacrolimus levels. (XLSX)

S3 Table Low dose animals blood (Group 3) tacrolimus levels. (XLSX)

S4 Table Rejection grade by time, all groups. (XLSX)
